# The prevalence and bacterial distribution of peritonitis amongst adults undergoing continuous ambulatory peritoneal dialysis at Universitas hospital

**DOI:** 10.4102/sajid.v35i1.104

**Published:** 2020-03-16

**Authors:** Jolly Musoke, Feziwe Bisiwe, Akhil Natverlal, Ilyas Moola, Yusuf Moola, Umar Kajee, Antonio Parlato, Andrea Bailey, Jerome Arendse

**Affiliations:** 1 Department of Medical Microbiology, Faculty of Health Sciences, University of the Free State, Bloemfontein, South Africa; 2 Department of Internal Medicine, Faculty of Health Sciences, University of the Free State, Bloemfontein, South Africa; 3 Department of Biostatistics, Faculty of Health Sciences, University of the Free State, Bloemfontein, South Africa

**Keywords:** CAPD patients, peritonitis, Universitas Academic Hospital, bacterial distribution, peritoneal dialysis

## Abstract

**Background:**

Peritonitis is the leading cause of morbidity and technique failure in peritoneal dialysis (PD) patients. The International Society for Peritoneal Dialysis (ISPD) recommends each centre to monitor the peritonitis rates and the causative organisms in order to guide local empiric antibiotic protocols. The aim of this study was to report on the peritonitis rates and describe the causative microorganisms and the antibiotic susceptibility in continuous ambulatory peritoneal dialysis (CAPD) adult patients at the Universitas Academic Hospital.

**Methods:**

A single-centre, retrospective descriptive survey was conducted to determine the peritonitis rates in PD patients (January–December 2016). All CAPD patients aged ≥18 years, who presented with clinical features of PD-associated peritonitis, were included. The peritonitis episodes were studied per patient, and the causative microorganisms and the antibiotic susceptibility of the organisms were described.

**Results:**

One hundred and twenty-eight patients underwent CAPD. The peritonitis rate was 1.45 episodes per year at risk. The prevalence of CAPD patients affected by at least one episode of CAPD-associated peritonitis during 2016 was 56.3%. The majority of episodes (76.7%) (*n* = 122) were mono-microbial. Gram-positive organisms accounted for 73.0% (*n* = 116) of the peritonitis episodes, coagulase-negative *Staphylococcus* being the most common. Gram-negative organisms accounted for 15.7% (*n* = 25) of the peritonitis episodes, and the common pathogens was Enterobacteriaceae.

**Conclusion:**

The peritonitis rate was alarmingly high, with 1.45 episodes per year at risk; this is three times more than the recommended 0.5 episodes per year according to the ISPD guidelines. The culture-negative rate of 8.8% is within ISPD-acceptable limits. There is a need to strengthen preventive measures with regard to peritonitis.

## Introduction

The increasing prevalence of chronic kidney disease (CKD) remains a worldwide public health problem, especially in developing countries.^1^ Kidney transplantation is the preferred treatment modality for patients with end-stage kidney disease (ESKD); however, it is not widely available. Haemodialysis (HD) and peritoneal dialysis (PD) are the other two forms of renal replacement therapy, mostly utilised as either lifelong renal replacement therapies in patients who are not eligible for kidney transplantation or as bridging treatment for patients awaiting kidney transplantation.

In the South African public health care sector, renal replacement therapy is rationed because of limited resources and limited HD slots. At the Universitas Academic Hospital (UAH) and the rest of the Free State Province hospitals, the PD-first rule applies. This means that the patients who are potentially transplantable and therefore eligible for renal replacement therapy programme only have access to PD as their initial treatment modality, unless there is a compelling contraindication as judged by the panel. Patients who are sometimes not keen to have continuous ambulatory peritoneal dialysis (CAPD) as their treatment modality are selected, and enrolment of patients with suboptimal socio-economic background onto CAPD may predispose them to a high risk of PD-associated peritonitis.

Peritonitis remains the most common complication in PD patients, contributing significantly to mortality and morbidity.^2^ Peritonitis can be defined as ‘the inflammation of the serosal membrane that lines the abdominal cavity and the organs confined therein’.^3^ Gram-positive organisms such as *Staphylococcus epidermidis* and other normal skin flora are amongst the most common causes of bacterial peritonitis.^4^ It may occur because of contamination of the PD catheter (touch contamination) or translocation of bacteria from the gut or from haematogenous spread of infections.^5^ Knowledge of local susceptibility data is important to guide a protocol-driven approach to empiric antimicrobial therapy.

Over the past two decades, the rate of CAPD-associated peritonitis has significantly reduced in most parts of the world. Between the early 1990s and the early 2000s, the incidence of peritonitis in Hong Kong was reported to have declined to 0.46 episodes from 1.10 episodes per year.^6^ Just after 2000, various centres have reported a peritonitis rate of 0.2–0.6 episodes per year of treatment, which translates to about one episode in 20–60 patient-months, which is a significant improvement.^7^ However, in South Africa, higher rates have been reported, with a study conducted by Isla et al.^8^ reporting an overall peritonitis rate of 0.82 per year with 1-, 2- and 5-year patient survival rates of 86.7%, 78.7% and 65.3%, respectively.^8^

Peritonitis associated with peritoneal dialysis, as well as microbial susceptibility patterns have not yet been described for the CAPD patient population being treated at UAH.

This is a retrospective audit aimed to describe the local peritonitis rate as well as the microbiological profiles of causative organisms in order to guide local antimicrobial management protocols.

## Methods

### Study area and design

This study was conducted at the CAPD clinic located in UAH in Bloemfontein, Free State, South Africa. This is a tertiary hospital with a capacity of 636 beds and all CAPD patients from the Free State province state sector are managed in the hospital’s nephrology clinic.

The research was conducted as a retrospective, descriptive review of microbiological and medical records of CAPD patients who had CAPD-associated peritonitis in 2016 (January–December) as well as the causative bacterial species for peritonitis.

### Study population and data extraction

The study population included adult patients (aged 18 years or older) who were on CAPD treatment and had been diagnosed with CAPD-associated peritonitis at the UAH during 2016 (January–December). Peritonitis was diagnosed when a patient met at least two of the following criteria: (1) clinical features consistent with peritonitis, that is, abdominal pain plus cloudy dialysis effluent; (2) dialysis effluent white cell count (WCC) ˃ 100 µL/with over 50% polymorphs in the differential count; and (3) identification of infective organisms from the dialysis effluent using Gram stain or culture.^9^ The microbial analysis data of the PD effluent sent for both WCCs and microscopy, culture and sensitivity (MC&S) testing were extracted from the National Health Laboratory Service (NHLS) capturing system, TrakCare. Medical records from clinic files and electronic notes were used to obtain clinical and demographic information. The data were collected and entered into an Excel spreadsheet after each patient had been allocated a numerical code to ensure confidentiality. The data extracted included demographical data such as age and gender. Microbial and clinical information included PD effluent WCC, number of peritonitis episodes, the causative bacterial species for peritonitis and antibiotic susceptibility profiles of all.

**FIGURE 1:**
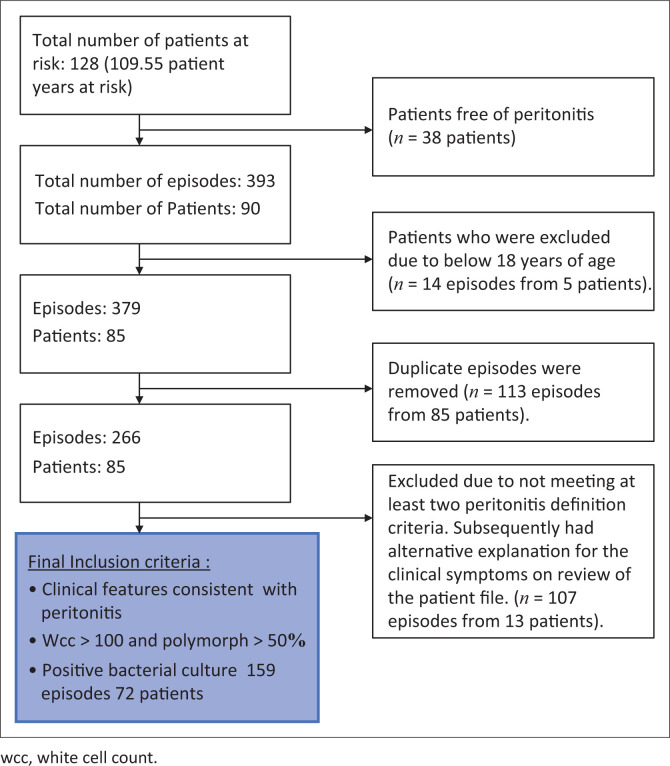
Flow diagram showing exclusion criteria.

The PD effluent was typically inoculated into a blood culture bottle for better yield. All microbiological results that did not fulfil the inclusion criteria were excluded (Figure 1).

In this study, peritonitis rates were defined according to ISPD guidelines that defined peritonitis rates as the number of infections by an organism for a time period, divided by dialysis years’ time at risk, expressed as episodes per year.^9^ The prevalence of peritonitis in CAPD patients affected by at least one episode of CAPD-associated peritonitis during the study period (January–December 2016) was calculated by dividing the number of patients who experienced at least one episode by the total number of patients. A relapsing case was defined as an episode that occurs within 4 weeks of completion of therapy of a prior episode with the same organism. The patient laboratory data were correlated with clinical records to confirm the clinical presentation of patients. Exclusion criteria entailed patients who did not meet the full diagnostic criteria defined above, and duplicate entries. ‘Duplicate entries’ was defined as having a specimen collected within 3 days and having the same causative organism. Culture-negative peritonitis episode refers to the patients that presented with clinical features suggestive of peritonitis (i.e. abdominal pain plus cloudy dialysis effluent) with a dialysis effluent WCC ˃ 100 µL/with over 50% polymorphs in the differential count but no growth on culture and Gram stain.

Statistical analysis was conducted by the Department of Biostatistics of the University of Free State using SAS 9.3 for Windows Software. Categorical variables were described as frequencies and percentages. Numerical variables were summarised by medians and interquartile ranges owing to skew distributions.

### Ethical consideration

Ethical approval was obtained from the Free State Department of Health, the National Health Laboratory Services and the University of the Free State (UFS-HSD2017/0429). All the personnel involved in the study handled all data and medical records with the highest confidentiality

## Results

During the year 2016 (from January to December), 128 patients underwent CAPD at UAH.

The median age of this study’s population was 41 (IQR 19–63).

Of the 128 patients, 38 patients were peritonitis free and 18 did not meet the inclusion criteria. A total of 72 patients presented with clinical features suggestive of CAPD-associated peritonitis and confirmed laboratory evidence and therefore met the inclusion criteria (see Figure 1). Of the 72 patients, 44.4% were men (*n* = 32) and 55.6% were women (*n* = 40).

The peritonitis rate was 1.45 episodes per year at risk, whereas the prevalence of CAPD patients affected by at least one episode of CAPD-associated peritonitis during 2016 was 56.3% ([72/128] × 100).

Majority of microbial infections were mono-microbial (Table 1).

**TABLE 1:** Classification of microbial infections.

Type of peritonitis	Number of episodes	%
Mono-microbial	122	76.7
Poly-microbial	19	12.0
Culture-negative	14	8.8
Fungal peritonitis	4	2.5

**Total**	**159**	**100**

We identified 159 episodes of peritonitis that ranged between one and six episodes per patient (Table 2), with 16.7% (12 patients) relapsing at least once. Only three patients relapsed twice (three episodes of the same organism in 2016).

**TABLE 2:** The number of episodes per patient.

Number of cases per year	One episode	Two episodes	Three episodes	Four episodes	Five episodes	Six episodes	Total (159 episodes)
Patients	31	17	10	8	4	2	72

## Microbial distribution

Most infections were caused by Gram-positive bacterial species (Table 3), the most common (39%) being coagulase-negative staphylococci (CNS). Amongst the identified CNS isolates, *Staphylococcus epidermidis* was the most prevalent species followed by *Staphylococcus haemolyticus, Staphylococcus hominis* and *Staphylococcus lugdunensis*.

**TABLE 3:** Bacterial distribution of aetiological organisms found in peritonitis (*n* = 159 episodes).

Type of microorganism	*n*	%
Gram-negative microorganisms	25	15.7
• *Klebsiella* species	10	6.3
• Escherichia coli	8	5.0
• Neisseria	5	1.3
• Others†	2	3.1
Gram-positive microorganisms	116	73.0
• Coagulase negative staph	62	39.0
• Coagulase positive staph	15	9.4
• *Streptococcus* species	20	12.6
• *Bacillus* species	16	10.1
• Others‡	3	1.9
Fungi	4	2.5
Culture-negative	14	8.8

† , *Acinetobacter baumannii, Proteus mirabilis* and *Chryseobacterium indologenes*;

‡ , *Enterococcus faecalis, Leuconostoc* species and *Corynebacterium jeikeium*.

Of the coagulase-positive staphylococci, *Staphylococcus aureus* was the most prevalent. Lastly *Streptococcus mitis oralis* (2.5%) was the leading cause of peritonitis amongst the *Streptococcus* species.

*Klebsiella* spp., *Escherichia coli* and *Neisseria* were the most common of the Gram-negative organisms, with a prevalence of 6.3%, 5.0% and 1.3%, respectively.

Four episodes were caused by the *Candida* species.

The culture negativity rate was 8.8% (*n* = 14).

## Antibiotic resistance

Cloxacillin and clindamycin showed resistance rates of 48.9% and 70%, respectively, for tested Gram-positive organisms, mostly CNS. This indicates high levels of methicillin resistance. No vancomycin-resistant episode (VRE) isolates were detected in the 12-month period. Amikacin was not routinely tested in the Gram-negative isolates; however, no resistance was detected for the ones that were tested.

## Patient outcomes

During the study period, 14% (*n* = 10) patients were transferred to haemodialysis. Seven patients (10%) died, but the cause of death was not documented. During the time that the study was undertaken (in 2016), 76% (*n* = 55) of the patients were still alive and were undergoing CAPD at the end of the year.

## Discussion

This study reported a peritonitis rate of 1.45 episodes per year at risk, which is three times higher than the recommended ISPD guidelines that state that peritonitis rates should not exceed 0.5 episodes per year at risk.^9^ This is of great concern and the possible reasons are multifactorial. This centre exercises a ‘PD-first’ rule, which often implicates that CAPD is conducted against the patient’s choice. This is a well-described risk factor for CAPD-associated peritonitis.^10^ The majority of patients in the Free State province present as crash landers and receive haemodialysis as an emergency treatment before starting CAPD. Haemodialysis prior to PD is a risk factor for developing peritonitis in the patient who later switches to CAPD.^10^ The majority of the patients also live far from the PD unit; on an average, patients travel 150 km to the centre with the furthest patient living 350 km from the dialysis centre; these patients often come from poor socio-economic backgrounds, which is another potential risk factor.^11^ The prevalence of CAPD patients affected by at least one episode of CAPD-associated peritonitis during 2016 in UAH was 56.3%. This is alarmingly high; however, this finding is consistent with findings from other centres in South Africa. In Durban, Ikabu et al.^12^ observed a prevalence of 49%, whereas in Cape Town, Raaijmakers et al.^13^ observed a prevalence of 71.6%.

The most common causative organism was CNS. This finding is in keeping with global trends, and it is most likely explained by the fact that CNS are skin colonisers and therefore easy contaminants during the PD process if the strict sterile method is breached.^4^ *Staphylococcus epidermidis* was the most prevalent of the CNS speciated. It is reassuring to note that all Gram-positive organisms were sensitive to vancomycin as this is the empiric antibiotic of choice in this centre; however, the rate of growth of methicillin-resistant CNS is concerning. This finding suggests that vancomycin is still an appropriate empiric antibiotic of choice to cover most Gram-positive organisms. It is, however, concerning that the wide use of vancomycin may predispose to vancomycin resistance. The patients who have peritonitis owing to methicillin-sensitive staphylococci are de-escalated to cefazolin upon availability of culture and sensitivity results.

Gram-negative organisms are known to be gastrointestinal contaminants and can commonly cause peritonitis by translocation from the gut into the peritoneal space. They are known to be associated with prolonged hospitalisation and higher risk of technique failure and increased mortality, compared to Gram-positive peritonitis.^14^ *Klebsiella* species was the most common Gram-negative bacteria followed by *E. coli.* In this study, Gram-negative organisms accounted for 15.7% of all peritonitis episodes. It is reassuring to note that none of the Enterobacteriaceae was either extended spectrum beta lactamase (ESBL) producing or carbapenem resistant.

A higher number of females were affected, with a prevalence of 55.6%. This study was not powered to evaluate risk factors for CAPD peritonitis; therefore, it is not possible to explain as to why females were more affected than males with peritonitis in this study population. These findings are however in accordance with the findings of Ros et al.^15^ who conducted the study based on information from the Andalusian transplant autonomic coordination registry and found that females experienced significantly higher infection-related mortality rate.^15^ Hypothetical explanations could include the increased risk of ascending Gram-negative infections from the female genitourinary tract or impaired immune response resulting from uremic hypogonadism.^16^

The culture negativity rate amounted to 8.8% (*n* = 14). This is lower than the ISPD standard, which stated that <10% of all episodes of PD peritonitis should be culture-negative.^9^ This success could possibly be attributed to the improved technique of inoculating PD effluent into the blood culture bottles instead of just sending a small sample in a specimen bottle as is routinely done for other body fluid samples.

Fungal peritonitis accounted for 2.5% of cases, and these were mostly *Candida* species. This is concerning, as fungal peritonitis is an indication to remove the PD catheter and therefore most often a result of technique failure. In a setting as in this study, where HD slots are limited, there is often a crisis to find haemodialysis slots.

There were no cases of tuberculous CAPD-associated peritonitis during the study period. This is interesting to note, as tuberculosis (TB) is endemic in South Africa, and the dialysis population has been identified as one of the populations at risk owing to an impaired immune system. This calls for a more targeted testing so as to ensure that cases are not missed out, as TB culture requires specific samples to be inoculated in the specific TB culture medium.

The number of patients who had relapsed infections was high. This is a cause for concern. Possible reasons for the relapses could be pharmacodynamics and pharmacokinetics that are altered by ESKD and inaccurate dosing, which may lead to sub-therapeutic drug levels.^17^ Intra-abdominal fluid locations can also impair the penetrance of the antibiotics and therefore increase the risk of relapse.^18^

## Limitation

This study did not investigate the risks for peritonitis.

## Conclusion and recommendations

The most common cause of infections in this study were CNS bacteria. No vancomycin-resistant organisms were detected; thus, vancomycin is still a drug of choice. Clindamycin and cloxacillin were less effective as there was high resistance to these drugs. We recommend that preventative measures be revisited such as developing more intense programmes for educating patients on hygiene; re-training patients when using the dialysis; evaluating PD programmes regularly; and comparing outcomes with international and local standards. We also recommend that the government officials in charge of housing consider prioritising these patients for placement in more suitable living conditions with access to clean water and optimal space where possible. We recommend an ongoing annual peritonitis registry to monitor the peritonitis trends and emergence of resistant organisms. More research is needed to study the risk factors of CAPD-associated peritonitis in this population so that more targeted preventive measures can be devised.
